# Iron accumulation and partitioning in hydroponically grown wild and cultivated chickpea (*Cicer arietinum* L)

**DOI:** 10.3389/fpls.2023.1092493

**Published:** 2023-03-17

**Authors:** Tamanna A. Jahan, Shweta Kalve, Zachery Belak, Christopher Eskiw, Bunyamin Tar’an

**Affiliations:** ^1^ Department of Plant Sciences, College of Agriculture and Bioresources, University of Saskatchewan, Saskatoon, SK, Canada; ^2^ Department of Food and Bioproduct Sciences, College of Agriculture and Bioresources, University of Saskatchewan, Saskatoon, SK, Canada

**Keywords:** Fe accumulation, Fe translocation, gene expression, chickpea, biofortification

## Abstract

Chickpea (*Cicer arietinum* L.) is a staple food in many developing countries where iron (Fe) deficiency often occurs in their population. The crop is a good source of protein, vitamins, and micronutrients. Fe biofortification in chickpea can be part of long-term strategy to enhance Fe intake in human diet to help to alleviate Fe deficiency. To develop cultivars with high Fe concentration in seeds, understanding the mechanisms of absorption and translocation of Fe into the seeds is critical. An experiment was conducted using a hydroponic system to evaluate Fe accumulation in seeds and other organs at different growth stages of selected genotypes of cultivated and wild relatives of chickpea. Plants were grown in media with Fe zero and Fe added conditions. Six chickpea genotypes were grown and harvested at six different growth stages: V3, V10, R2, R5, R6, and RH for analysis of Fe concentration in roots, stems, leaves, and seeds. The relative expression of genes related to Fe-metabolism including *FRO2*, *IRT1*, *NRAMP3*, *V1T1*, *YSL1*, *FER3*, *GCN2*, and *WEE1* was analyzed. The results showed that the highest and lowest accumulation of Fe throughout the plant growth stages were found in the roots and stems, respectively. Results of gene expression analysis confirmed that the *FRO2* and *IRT1* were involved in Fe uptake in chickpeas and expressed more in roots under Fe added condition. All transporter genes: *NRAMP3*, *V1T1*, *YSL1* along with storage gene *FER3* showed higher expression in leaves. In contrast, candidate gene *WEE1* for Fe metabolism expressed more in roots under Fe affluent condition; however, *GCN2* showed over-expression in roots under Fe zero condition. Current finding will contribute to better understanding of Fe translocation and metabolism in chickpea. This knowledge can further be used to develop chickpea varieties with high Fe in seeds.

## 1 Introduction

Globally, the population is increasing and is projected to reach 9.1 billion by 2050. As a result, around 70% higher demand for food is needed during the same period (WSFS, 2009). This situation challenges support the rapid growth of the global economy. Although added food production has been generated through high yielding cultivars of staple crops, hundreds of millions of people still suffer from micronutrient deficiency (Satterthwaite et al., 2010). Among a variety of micronutrient deficiencies, Fe deficiency is one of the most common and extensive malnutrition world-wide (Baltussen et al., 2004). Globally, Fe deficiency is considered the 6^th^ highest cause of mortality and top 10 health challenges in present day (Briat, 2011; Campion et al., 2013). Fe deficiency may occur throughout a lifetime if the diets are mainly based on cereals and legumes (WHO, 2002). Anemia, which is mainly due to Fe deficiency, affects around 2 billion people in the world (Briat, 2011). By increasing the amount of Fe in the diet, Fe deficiency can be overcome (WHO, 2005). However, solving Fe deficiency in developing countries is difficult as the population relies mostly on staple food crops as sources of micronutrients which are inherently low in Fe (Gómez-Galera et al., 2010). To address this problem, micronutrient dense cultivars with high yielding capacity are needed (Zhu et al., 2007). Chickpea is an important legume crop annually grown over 14 million hectares in 59 countries around the globe. It is an inexpensive, high-quality source of protein along with vitamins and minerals including Fe. In many countries such as India and the Middle East, chickpea is a staple food crop and a major component of the diets (Zhu et al., 2005; Millán et al., 2015). Chickpea consumption has been rising in many countries around the world (Tan et al., 2017). As such, chickpea has a potential to serve as a vehicle to address Fe deficiency in human. Chickpea cultivars with high Fe concentration in seeds are needed.

Fe is commonly found in an abundant amount in the earth’s crust; however, its limited solubility results in limited uptake by plants. Consequently, only low amount of Fe is accumulated in the edible parts of the plants (Zuo and Zhang, 2011). Besides the uptake of Fe from soil to the roots, Fe accumulation in the seeds depends on the translocation of Fe to the vegetative tissues and loading it into the seeds. Fe concentration in plant organs also varies based on the species and cultivars. Plants’ ability to acquire and accumulate Fe in different tissues is under genetic Fe zero (Sankaran and Grusak, 2014). Fe deficiency in plants will result in low levels of Fe in the seeds that ultimately affect human nutrition (Zuo and Zhang, 2011).

One strategy to mitigate Fe deficiency in human population is to increase Fe concentration and its bioavailability in the edible part of the plants (Briat, 2011). Fe biofortification is a long-term approach to improve Fe nutrition. To reach this goal, genetic modifications and plant breeding offer a possibility for improving Fe amount and bioavailability of crops (Briat, 2011).

In the efforts toward generating Fe biofortified crops, several genes associated with Fe metabolism in plants have been identified in different species. In nongraminaceous plant species, the dominant genes responsible for Fe uptake are *FRO2 (Ferric-chelate reductase oxidase gene)* and *IRT1 (Fe-regulated transporter gene)*. These two-uptake genes strongly responded under Fe zero environment (Eide et al., 1996; Robinson et al., 1999). Genes responsible for Fe translocation such as *YELLOW STRIPE 1-like (YSL)* gene family (Koike et al., 2004) has also been identified. *Natural resistance-associated macrophage protein 3 (NRAMP3)* and *vacuolar Fe transporter 1 (V1T1)* are two core genes that are involved in subcellular transportation of Fe (Thomine et al., 2000; Lanquar et al., 2005; Kim et al., 2006). *Ferritin (FER)* gene is responsible for high-capacity Fe storage and sequestration (Petit et al., 2001).

One obstacle to Fe biofortification is the lack of knowledge of how Fe is accumulated into the seeds (Sankaran and Grusak, 2014). As a result, uncertainty occurs when it comes to select the appropriate pathways or genes to target for selection or modifications in genetic improvement program. This problem supports the urgency to understand the mechanism of Fe partitioning from the roots to the seeds for breeding of Fe rich crops.

Using genetic transformation technique, Lee and An (2009) reported that overexpression of Fe acquisition gene *IRT1* increased the Fe concentration by 1.1-folds in brown rice grain. In rice and wheat, the overexpression of nicotinamine synthase (*NAS)* improved Fe concentration by up to 2-folds (Johnson et al., 2011; Beasley et al., 2019). Furthermore, the expression of *GmFERH1* gene increased Fe concentration up to 3-folds in both brown and polished rice. The overexpression of *TaVIT2* in wheat and *OsVIT1* or *OsVIT2* in rice increased Fe amount by 2 and 1.3-folds, respectively (Zhang et al., 2012; Connorton et al., 2017). Successful results also found by the introduction of transgene combination, such as the combined overexpression of *IRT1* and *PvFER1* increased up to 4-folds Fe in the endosperm of polished rice (Boonyaves et al., 2017). When the transgene combination of *PvFERRITIN*, *AtNAS1* and *Afphytase* expressed together resulting in a 6-folds increase in rice seed Fe concentration (Wirth et al., 2009). In field grown polished rice, another different multiple transgene combination (*HvNAS1*, *OsYSL2*, and *GmFERRITIN*) was reported to increase Fe amount by 4.4-folds (Masuda et al., 2013). Overexpression of nicotinamine synthase (*NAS*) gene resulted in increase nicotinamine (NA) amount in rice and wheat that ultimately increased Fe bioavailability in mice or Caco-2 cell culture model (Zheng et al., 2010; Beasley et al., 2019).

Information of Fe translocation, partitioning and accumulation to the seeds at different growth stages as well as the related genes associated with the process is needed to develop cultivars with high Fe concentration. To date, information on the dynamics of Fe accumulation in chickpea seeds is lacking. Therefore, the first step to increase Fe amount in chickpea seeds is to examine the mobilization of Fe accumulation in plant organs of diverse chickpea genotypes. The output of the study will help in the development of new breeding strategies to improve Fe concentration in chickpea seeds. The main objectives of this study were: 1) to evaluate Fe accumulation in organs at different growth stages of diverse chickpea genotypes, and 2) to evaluate the expression levels of genes associated with Fe uptake, transportation, and accumulation into the seeds of chickpea.

## 2 Materials and methods

The research was conducted in a Fe zeroled chamber at the College of Agriculture and Bioresources, University of Saskatchewan. The whole experiment was repeated twice with four replications each time. The chamber was adjusted with 16 h, 22°C, and 8 h, 15°C Day-night regime. The light intensity of PAR was 220 µmol m^-2^ s^-1^ of photon flux density. Light was provided with a combination of florescent tubes and incandescent bulbs.

### 2.1 Plant materials

**Figure 1:**
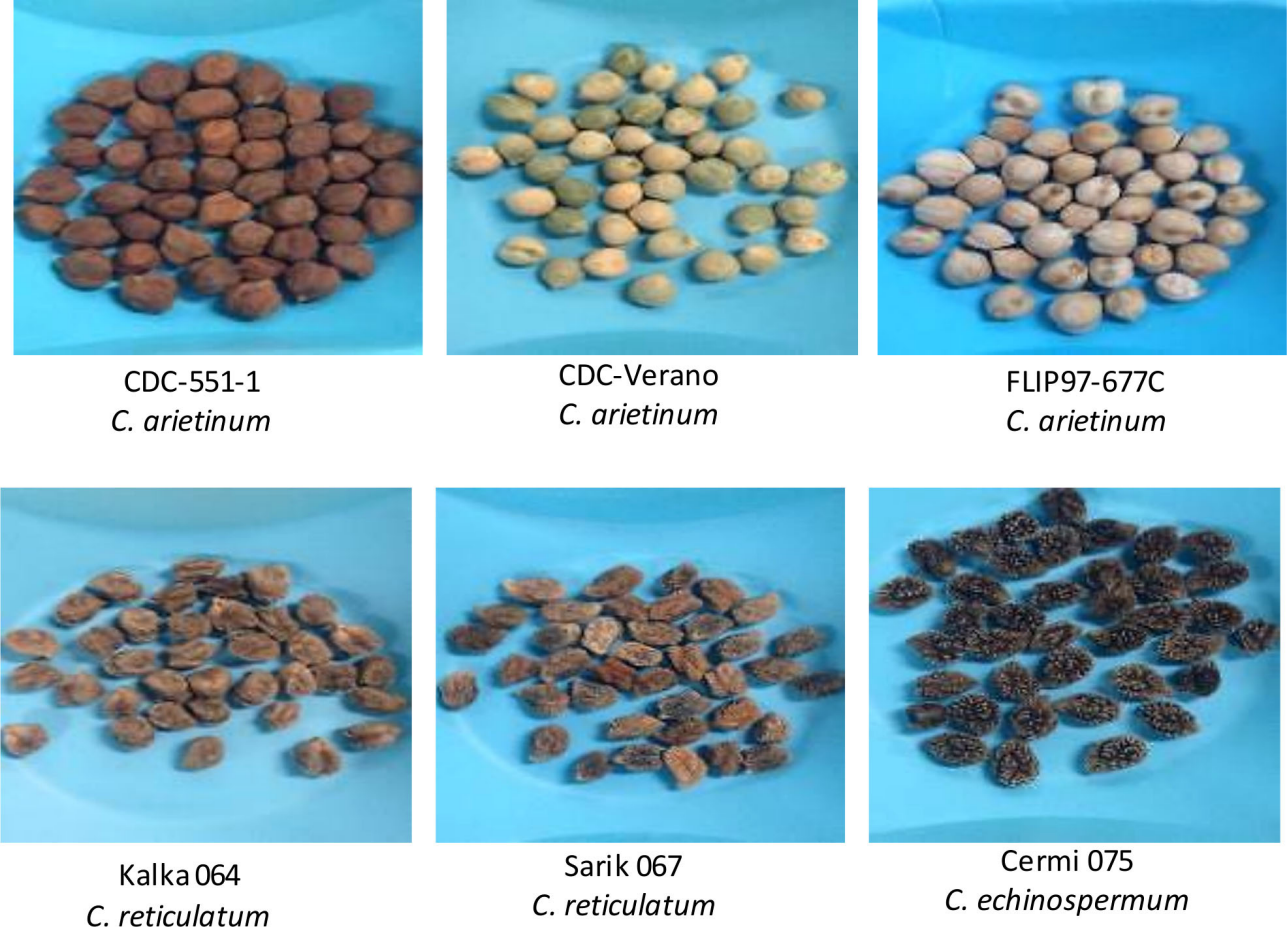
Six diverse chickpea genotypes used for the study of Fe absorption and accumulation in the chickpea.

Six chickpea genotypes, namely CDC-551-1, CDC Verano, FLIP97-677C, Kalka 064, Sarik 067, and Cermi 075 were used in this study (**Figure 1**).

These genotypes were collected from the chickpea breeding program at the Crop Development Centre, University of Saskatchewan. Three genotypes (**Figure 1**) belong to the cultivated species (*Cicer arietinum*: CDC Verano, FLIP97-677C, and CDC-551-1), and the other three are wild species accession *(C. reticulatum*: Sarik 067 and Kalka 064 and *C. echinospermum*: Cermi 075). Prior analysis from field grown plants showed that these six genotypes had varying Fe concentrations in seeds (**Table 1**).

**Table 1:** Description of six chickpea genotypes with the seed size (g/1000seeds) and mean Fe concentration in seeds (µg g^-1^ ± SE) evaluated in the study of Fe absorption and accumulation in different organs.

No.	Name	Species	Type	Status	Seed size (g/1000 seeds)	Average Fe conc. in seeds (µg g^-1^ ± SE)
1.	CDC-551-1	*Cicer arietinum*	Desi	Breeding line	297	41.6 ± 0.8
2.	CDC Verano	*Cicer arietinum*	Kabuli	Cultivar	165	60.1 ± 0.4
3.	FLIP97-677C	*Cicer arietinum*	Kabuli	Breeding line	396	52.4 ± 0.4
4.	Kalka 064	*Cicer reticulatum*	Wild species	Germplasm	143	49.3 ± 0.3
5.	Sarik 067	*Cicer reticulatum*	Wild species	Germplasm	164	95.2 ± 0.8
6.	Cermi 075	*Cicer echinospermum*	Wild species	Germplasm	149	40.3 ± 0.2

Source of information and initial Fe concentration: Diapari et al., 2014; Mengistu (2016).

Plants were grown using hydroponic system in the phytotron chamber. Seeds were pre-germinated before transferring them to polyethylene containers with nutrient solution using hydroponic system (Grusak et al., 1990). Only plants from V3(3^rd^ multifoliate leaf has unfolded from the stem) and V10 (10^th^ multifoliate leaf has unfolded from the stem) growth stage were grown in both Fe added (Fe^+^, 5 μM Fe (III)-EDDHA [ethylenediamine- *N*, N bis(*o*-hydroxyphenyl) acetic acid] and Fe zero [Fe^-^, 0 μM Fe (III)-EDDHA] conditions. The other stages were only grown under Fe added condition to skip the chlorosis. The volume of the nutrient mixture was adjusted according to the number of plants grown in each container. For the vegetative stages (V3 and V10), twelve plants of each genotype were grown in each container. However, for the reproductive stages R2 (full bloom stage), R5 (early seed stage) and R6 (full seed stage) and full maturity stage (RH), four plants were grown at equal space on the container to minimize the tangle of roots. Plant samples were collected from the 2^nd^ week at the 3^rd^ node stage (first multifoliate leaf stage) to physiological maturity.

### 2.2 Media preparation

The hydroponically grown plants were provided with nutrient solution containing the following macronutrients: 3.6mM KNO_3_, 2.4mM Ca(NO_3_)_2_, 0.3mM NH_4_H_2_PO_4_, 0.6mM MgSO_4_, 75µM CaCl_2_, 75µM H_3_BO_3_, 6µM MnSO_4_, 6µM ZnSO4, 1.5µM CuSO_4_, 1.5µM H_2_MoO_4_, and 0.3µM NiSO_4_, and was buffered with 3mM MES[2-(N-morpholino) ethane sulfonic acid] to maintain the pH between 5.5-6.0. Another buffer, pH down^©^, was used to lower the pH if needed. Fe solution was prepared according to Chaney and Bell (1987). Fe was added as 5µM Fe (III)-EDDHA per four plants according to Grusak (1994).

### 2.3 Experimental design

The Fe concentration, Fe amount and gene expression analysis in different organs of six different chickpea plants was done with four replications with two repeats in both Fe added and Fe zero conditions. In this study, seven growth stages: two vegetative stages (V3 = 3^rd^ multifoliate leaf has unfolded, and V10 = full vegetative stage), three reproductive stages (R2 = full bloom, R5 = early seed, and R6 = fully developed seed) and one physiological maturity stage (RH = 90% of pods are golden brown) were used for measuring the Fe concentration (µg g^-1^). Fe analysis was done for each organ separately including roots, stems, leaves, and mature seeds. Roots, stems, and leaves at the vegetative stage, and seeds at the reproductive and maturity stage were digested and prepared for Fe analysis using inductively couple plasma (ICP) –atomic emission spectrometry (iCAP 6500 series: Thermo Jarrell Ash Corp., Franklin, MA, USA) at plant biochemistry and molecular physiology lab, University of Saskatchewan. However, for gene expression analyses, only two vegetative stages (V3 = 3^rd^ multifoliate leaf has unfolded, and V10 = full vegetative stage) and two reproductive growth stages (R2 = full bloom, and R5 = early seed) were used. The gene expression analysis was done in root and leaf using qPCR. For growth stages R2, R5, R6, and RH, one plant was harvested for each replication. However, at V3 growth stage, to get 0.5g tissue sample, six plants were harvested for each replication for measuring Fe concentration (µg g^-1^) and gene expression analyses. For V10, three plants were harvested for each replication. For dry weight in roots, shoots, and roots to shoots (dry weight/dry weight) ratio, six and three plants were harvested for each replication for measuring root and shoot dry weight at V3 and V10 growth stages, respectively.

### 2.4 Data collection

#### 2.4.1 Fe concentrations

Samples were prepared from roots, stems, leaves, and seeds. From V3 and V10 vegetative growth stages, three samples: roots, stems, and leaves were collected. Seeds were collected from the reproductive growth stages R5 and R6 as well as the physiological maturity growth stage (RH) along with roots, stems, and leaves. Pods were tagged by using different colored threads to ensure appropriate growth stage for collecting seeds. For the R5 growth stage, pods were harvested at 16 days after anthesis. However, for R6 and RH growth stages, pods were harvested at 24 and 32 days after anthesis. For each stage around 30 pods were harvested to get enough tissue samples. Roots were collected at each growth stage. Roots were rinsed (2.5 min each rinse) twice with aerated deionized water. The root samples were blotted dry and were placed in paper bags for oven drying and subsequent dry weight determination. Besides V3 and V10, per time point, a total of 4 plants were analyzed. All tissue samples were dried at 60°C followed by weighing. After weighing, each tissue samples were transferred to polycarbonate tubes and homogenized with geno grinder (SPEX™ SamplePrep, 65 Liberty Street, Metuchen, NJ) to get the powdered samples (Waters and Grusak, 2008). Each sample was digested by taking 0.5g of dried tissue sample using 4mL of concentrated nitric acid and 2mL of perchloric acid at 200°C temperature followed by drying. Digests were suspended again in 1mL of 2M HNO_3_ and after 1h was brought to 10mL with deionized water. The acids used were as trace metal grade (Fisher Scientific, Pittsburgh, Pennsylvania, USA) and the water was deionized *via* a MilliQ system (Millipore, Billerica, Massachusetts, USA). Samples were then analyzed for Fe concentration by using inductively couple plasma –atomic emission spectrometry. Fe amount from each tissue was calculated by multiplying the average Fe concentration from each tissue by the average tissue weight per plant at a given time point. Fe amount, root and shoot dry weight were measured from the average tissue weight of six and three plants at V3 and V10 vegetative growth stages, respectively.

#### 2.4.2 Gene expression analysis

Young root and leaf samples were harvested and immediately put in liquid nitrogen and stored at -80 ° C until RNA extraction. Tissue samples were ground using sterilized mortar and pestle before RNA extraction. RNA was extracted and treated with DNase I using Qiagen RNeasy plant mini kit (Qiagen, Valencia, CA, USA). Thereafter, RNA quantity and purity was measured using an optical density reading at 260nm and the 260/280 and the 260/230 absorption ratios using NanoDrop 800 UV-vis spectrophotometer (Thermo Fisher Scientific Inc, USA). RNA integrity was measured on 1% agarose gel using MOPS buffer (3-morpholinopropane-1-sulfonic acid). 1 µg of total RNA was reverse transcribed to cDNA using SensiFast cDNA synthesis kit (Bioline, Inc.). Before running qPCR, cDNA was diluted 5X with DNase/RNase free water to get the specific amount of cDNA according to the manufacturer instructions (applied biosystem qPCR protocol). Primers were designed for each of the selected genes and the reference gene *GAPDH* by using IDT primer quest tool (IDT, 2018). *GAPDH* was selected and used as internal Fe zero to normalize the relative quantities of the target genes due to its consistency across the different growth stages and genotypes. The reverse and forward primer sequences are presented in **Table 2**.

**Table 2:** Name and general function of selected genes involved in Fe metabolism along with the forward and reverse primer sequences used in qPCR analysis.

Gene	General Function	Primer sequence
*GAPDH*	Reference Genes	F 5’- CCAAGGTCAAGATCGGAATCA -3’ R 5’- CAAAGCCACTCTAGCAACCAAA -3’
*FRO2*	Fe Root Uptake	F 5’- CTGCAGAGGATGGCGATAAA -3’ R 5’- GAACCACGAGTCACTGGAAA -3’
*IRT1*	Fe Root uptake	F 5’- GCTTTCGCTTCTGGTGTTATAC -3’ R 5’- CCAAGGACGCTGAGGTAAA -3’
*NRAMP3*	Fe Transport	F 5’- CACGGCTATGGGACTTCTTATT-3’ R 5’- TCCTAGCCCAACTAGGATACTC-3’
*V1T1*	Fe Transport	F 5’- GAGAAACCAGATCCAAGGAGAG -3’ R 5’- GGAATGAACGCGTAAGGAATG -3’
*YSL1*	Fe Transport	F 5’- GTGTGGTAGCAGGACTTGTAG -3’ R 5’- CGGAGAGGTACGTGTGTAATG -3’
*FER3*	Fe Storage	F 5’- CCTATGTGTACCATTCCATGTTTG -3’ R 5’- ACTCTTCCACCACGATTGTTC -3’
*WEE1*	Fe Metabolism	F 5’- GCAAGTTGCCACTACTACCT -3’ R 5’- CTCTCTAGCCGAAGGTCTCTTA -3’
*GCN2*	Fe Metabolism	F 5’- ACGACAGTGAAGGTGAGAAAG -3’ R 5’- GATGAGGCAAGGAGACAGAAG -3’

Each primer pair was designed to span exon-exon junction with PCR product size 89 to 115 bp, length of primer sequence 20 to 22 nucleotides, temperature 61 to 62°C, and the GC content 47.6 to 50%. Before checking primer efficiencies of each selected and reference gene, cDNA was diluted 5X. The target gene expression analysis was done using SensiFAST SYBR No-ROX kit using optical 96 well plate on QuantStudio™ 3 Real-Time PCR System. The genomic DNA contamination or primer dimers was checked on PCR by using Fe zeros [negative reverse transcription Fe zero (-RTC)] and no template Fe zero (NTC)] for each genotype at each time.

After completing 40 amplification cycles, specificity of PCR product per gene was observed by analysing melting curve. All samples for each amplicon had a single sharp peak at the amplicon melting temperature. Genes involved in Fe metabolism, were selected for the analysis. Candidate genes: *FRO2* (García et al., 2012), *IRT1* (García et al., 2012), *NRAMP3* (Lanquar et al., 2010), *VIT1*(Brear et al., 2013)*, YSL1*(Kim et al., 2006), *FER3* (Briat et al., 2010), *WEE1* (Mendes, 2014) and *GCN2* (Mendes, 2014) were selected based on their crucial role in Fe metabolisms.

Gene expression analyses were completed using three biological replications along with two technical replications. The gene expression of each tissue sample at different growth stages was averaged over four replications with two repeats. The standard curve method was used for the absolute quantification of the expression of selected genes and the expression were calculated by 2^(-Δ^ *^CT^* ^)^ method (Schmittgen and Livak, 2008).

### 2.5 Statistical analysis

The Fe concentrations from different organs of the plants grown under Fe added and Fe zero conditions were averaged over four replications and two repeats. Fe amount from each tissue sample was calculated by the following formula:

*Fe amount (g) = Mean Fe concentration of organ (µg g^-1^) x Mean organ dry weight (g)/1000*

Descriptive statistics were used to calculate mean ± standard error (SE). The PROC GLM of SAS version 9.4 (SAS Institute Inc., Cary, NC, USA) was used to compare the means of roots and shoots dry weight and roots to shoots (dry weight/dry weight) ratio.

## 3 Results

### 3.1 Fe concentrations

**Figure 2:**
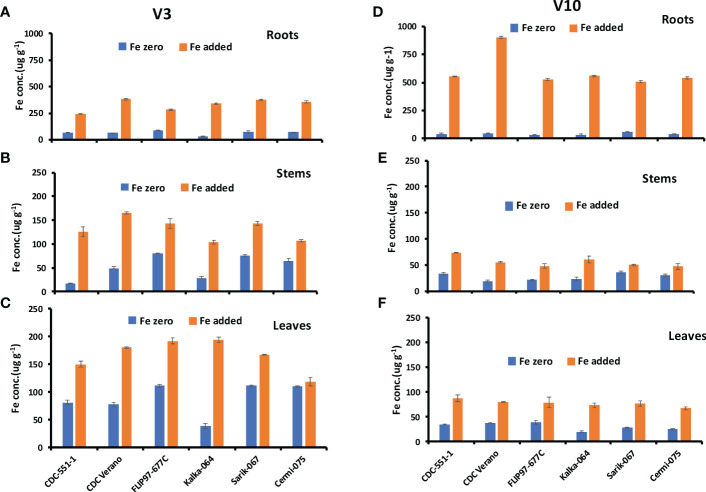
Mean Fe concentration (µg g^-1^, ± SE, n = 48) at V3 and mean Fe concentration (µg g^-1^, ± SE, n = 24) at V10 growth stages in roots **(A, D)**, stems **(B, E)**, and leaves **(C, F)** of six different genotypes of chickpea under Fe zero and Fe added conditions.

Fe concentrations in roots, stems, and leaves of six chickpea genotypes at V3 and V10 growth stages are presented in **Figure 2**.

The Fe concentration in roots of six genotypes at V3 and V10 growth stages are presented in **Figures 2A, D**. Results showed that Fe concentration in roots from plants grown under Fe zero (no Fe added) condition decreased from V3 to V10 growth stage in all six genotypes. The reduction rate of Fe concentration from V3 to V10 under Fe zero condition was higher in cultivated species (33%-62%) compared to wild species (6%-50%). In contrast, under Fe added condition, Fe concentration level increased From V3 to V10 growth stages (**Figures 2A, D**). Like in roots, Fe concentration levels in stem and leaf tissues in plants grown under Fe zero condition decreased from V3 to V10 growth stage in the selected genotypes, except the stems of CDC-551-1 (**Figures 2B, C, E, F**).

The present study also showed that at V3 growth stage, the highest Fe concentration level under Fe zero condition was observed in leaves followed by roots and stems. However, under Fe added condition, the highest Fe concentration level was observed in roots over six different growth stages (**Figures 2A, D**). As in the Fe zero environment, under Fe added condition, Fe in leaves decreased dramatically from V3 to V10 growth stages (**Figures 2C, F**). During the regenerative stage (R2 to RH), Fe concentration in roots increased with slight fluctuation at R6 and RH stages over six genotypes (**Supplementary Table S1**). However, Fe concentration in stems and leaves were relatively stable with slight variations across the genotypes (**Supplementary Tables S2 and S3**).

### 3.2 Fe amount

**Figure 3:**
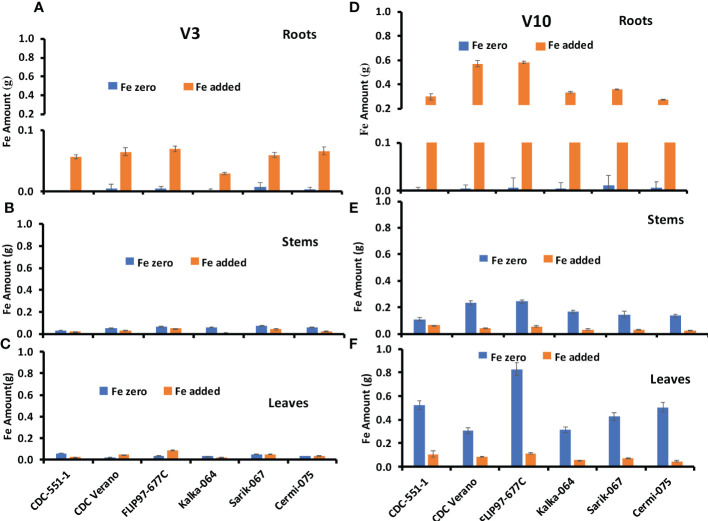
Mean Fe amount (g, ± SE, n = 48) at V3 and mean Fe amount (g, ± SE, n = 24) at V10 growth stages in roots **(A, D)**, stems **(B, E)**, and leaves **(C, F)**, of six different genotypes of chickpea under Fe zero and Fe added conditions.

Fe amount in leaves, stems, and roots of six chickpea genotypes at V3 and V10 growth stages are presented in **Figure 3**.

The Fe amount in roots of six genotypes at V3 and V10 growth stages are presented in **Figures 3A, D**. Results showed that Fe amount in roots from plants grown under Fe zero condition slightly increased from V3 to V10 growth stage in all six genotypes except CDC Verano. Likewise, Fe absorption and accumulation in roots increased from V3 to V10 growth stage under Fe added condition (**Figures 3A, D**). Similar observation was found in reproductive growth stage R2 to physiological maturity stage R6 (**Supplementary Table S4**).

The Fe amount in stems of six genotypes at V3 and V10 growth stages are presented in **Figures 3B, E**. Fe amount under Fe zero condition increased more from V3 to V10 growth stage compared to Fe added condition. Like in the roots, Fe amount in the stems showed higher level at the physiological maturity stage (**Supplementary Table S5**).

The Fe amount in leaves of six genotypes at V3 and V10 growth stages are presented in **Figures 3C, F**. As in stems, Fe amount under Fe zero condition increased more from V3 to V10 growth stage compared to Fe added condition. Similarly, the lowest Fe amount in leaves was found in the V3 growth stage compared to V10 under both conditions (**Figures 3C, F**). In contrast to the roots and stems, the Fe amount in leaves decreased sharply from the physiological maturity stage R6 to RH (**Supplementary Table S6**).

### 3.3 Fe concentrations vs Fe amount in seeds

**Figure 4:**
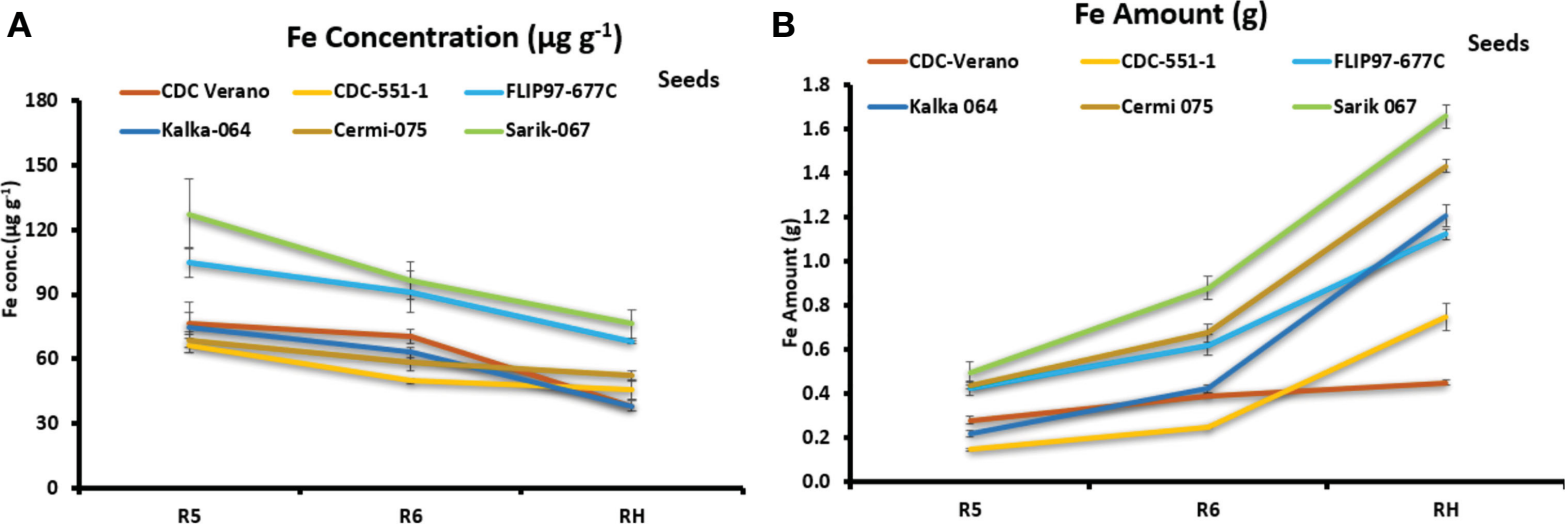
Mean Fe concentration (µg g^-1^, ± SE, n = 8) **(A)** and mean Fe amount (g total seeds^-1^ plant^-1^, ± SE, n = 8) **(B)** in seeds of six chickpea genotypes at R5, R6, and RH growth stages.

Fe partitioning in seeds showed that Fe concentration levels decreased gradually over three growth stages: R5, R6, and RH among the six genotypes (**Figure 4A**).

In contrast, data from three reproductive growth stages R5, R6, and RH showed that Fe amount in seeds increased gradually from R5 to RH. Genotypes Sarik 067 (1.7 g) and CDC Verano (0.5 g) showed the highest and the lowest Fe amount at RH stage, respectively (**Figure 4B**). Similarly, at RH stage, genotypes Sarik 067 had the highest Fe concentration (76 µg g^-1^) in seeds followed by FLIP97-677C (68 µg g^-1^), whereas Kalka 064 had the lowest (38 µg g^-1^) (**Figure 4A**). Our findings showed that Fe concentration level in seeds was higher in reproductive stages (R5 and R6) compared to full maturity stage (RH) (**Figure 4A**). However, Fe amount level in seeds was higher in full maturity stage (RH) compared to reproductive stages (R5 and R6) (**Figure 4B**).

### 3.4 Dry weight

Dry weight in roots, shoots, and roots to shoots ratio of six chickpea genotypes in Fe zero and Fe added conditions at V3 and V10 growth stages are presented in **Table 3**.

**Table 3:** Root to shoot (dry weight/dry weight) ratio of six chickpea genotypes under Fe zero and Fe added conditions at V3 and V10 growth stages.

Genotypes	Growth Stages	Growth Conditions	Roots DW (g ± SE)	Shoots DW (g ± SE)	Roots/shoots (DW/DW ± SE) ratio
CDC-551-1	V3	Fe zero	0.03 ± 0.00^a^	0.09 ± 0.00^a^	0.40 ± 0.03^def^
		Fe added	0.23 ± 0.01^f^	0.32 ± 0.01^b^	0.73 ± 0.02^h^
	V10	Fe zero	0.07 ± 0.00^ab^	0.63 ± 0.00^e^	0.11 ± 0.01^a^
		Fe added	0.83 ± 0.01^j^	2.06 ± 0.04^k^	0.41 ± 0.02^def^
CDC Verano	V3	Fe zero	0.08 ± 0.00^abd^	0.07 ± 0.00^a^	1.06 ± 0.08^g^
		Fe added	0.16 ± 0.01^bde^	0.44 ± 0.01^bc^	0.47 ± 0.05^eg^
	V10	Fe zero	0.10 ± 0.00^abcd^	0.54 ± 0.00^de^	0.19 ± 0.02^ab^
		Fe added	0.69 ± 0.01^i^	1.91 ± 0.00^j^	0.36 ± 0.01^d^
FLIP97-677C	V3	Fe zero	0.05 ± 0.01^a^	0.10 ± 0.00^a^	0.53 ± 0.01^g^
		Fe added	0.32 ± 0.00^l^	0.77 ± 0.01^f^	0.40 ± 0.02^def^
	V10	Fe zero	0.22 ± 0.00^ef^	1.07 ± 0.00^g^	0.21 ± 0.02^b^
		Fe added	1.04 ± 0.00^k^	2.55 ± 0.02^l^	0.41 ± 0.01^df^
Kalka-064	V3	Fe zero	0.06 ± 0.00^ab^	0.09 ± 0.04^a^	0.64 ± 0.01^h^
		Fe added	0.12 ± 0.01^cd^	0.19 ± 0.10^a^	0.65 ± 0.04^h^
	V10	Fe zero	0.15 ± 0.01^cde^	0.48 ± 0.03^cd^	0.32 ± 0.02^cd^
		Fe added	0.57 ± 0.02^h^	1.28 ± 0.02^h^	0.47 ± 0.02^eg^
Sarik-067	V3	Fe zero	0.11 ± 0.02^bcd^	0.12 ± 0.06^a^	0.89 ± 0.03^i^
		Fe added	0.12 ± 0.01^cd^	0.60 ± 0.05^de^	0.21 ± 0.01^b^
	V10	Fe zero	0.21 ± 0.01^bef^	0.57 ± 0.03^de^	0.66 ± 0.03^df^
		Fe added	0.71 ± 0.03^i^	1.55 ± 0.01^i^	0.46 ± 0.02^efg^
Cermi-075	V3	Fe zero	0.06 ± 0.02^ab^	0.09 ± 0.05^a^	0.66 ± 0.02^h^
		Fe added	0.17 ± 0.02^be^	0.52 ± 0.02^de^	0.34 ± 0.01^cd^
	V10	Fe zero	0.16 ± 0.01^cde^	0.64 ± 0.05^e^	0.24 ± 0.01^bc^
		Fe added	0.45 ± 0.02^g^	1.12 ± 0.03^g^	0.40 ± 0.02^def^

Data are means ± standard error (SE), n = 48 at V3, n = 24 at V10. Means in the same column with the different letters are significantly different based on LSD tests (p < 0.05). DW, Dry weight.

Results showed that the mean values for roots dry weight in Fe added conditions significantly higher (*p* < 0.05) than for the Fe zero conditions at both V3 and V10 stages in all six chickpea genotypes. The maximum and minimum mean value was observed in FLIP97-677C (1.04g) and CDC-551-1(0.03g) at V10 and V3 growth stages under Fe added and Fe zero conditions, respectively (**Table 3**). Like in roots dry weight, the mean values of shoots dry weight in Fe added condition also showed significantly higher (*p* < 0.05) than for the Fe zero conditions at both V3 and V10 growth stages except Kalka-064 at V3 stage. The highest and lowest mean score was observed in FLIP97-677C (2.55g) and CDC Verano (0.07g) at V10 and V3 growth stages under Fe added and Fe zero conditions, respectively (**Table 3**). Similarly, for roots to shoots (dry weight/dry weight) ratio, the mean values of roots to shoots ratio in Fe added condition also showed significantly higher ((*p* < 0.05) than for the Fe zero conditions at both V3 and V10 stages except Kalka-064 at V3 stage. The maximum average score for roots to shoots ratio was observed in CDC Verano (1.06) at V3 stage under Fe zero condition, whereas the lowest was observed in CDC-551-1(0.11) at V10 under Fe zero condition (**Table 3**).

### 3.5 Gene expression analysis

#### 3.5.1 V3 growth stage

**Figure 5:**
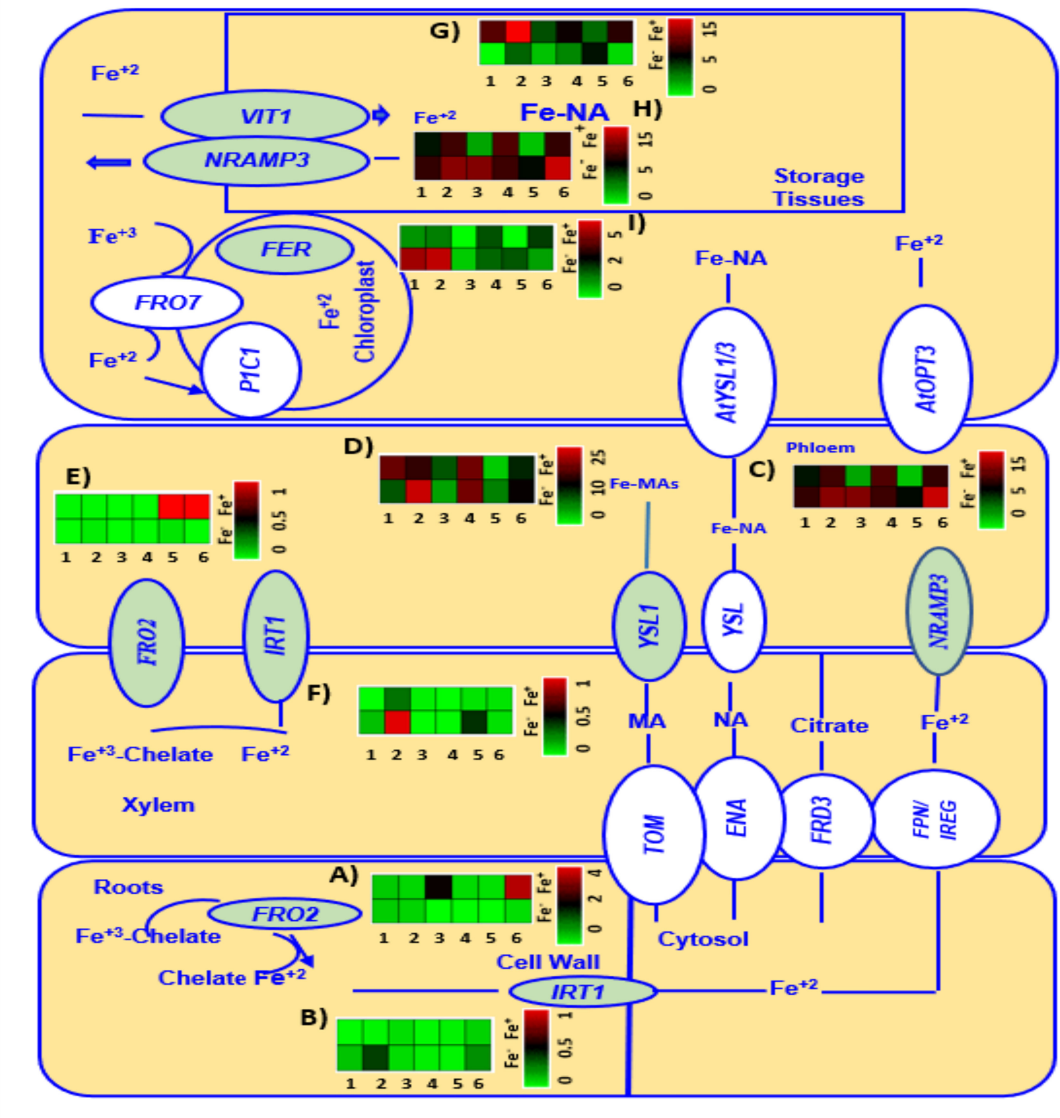
A heatmap analysis showing the gene expression patterns of Fe metabolism related genes *FRO2* **(A, E)**, *IRT1* **(B, F)**, *NRAMP3* **(C, H)**, *YSL1* **(D)**, *V1T1* **(G)**, and *FER3* **(I)** in roots and leaves of six genotypes (1 = CDC Verano, 2 = Cermi 075, 3 = FLIP97-677C, 4 = Sarik 067, 5 = Kalka 064, and 6 = CDC 551-1). The data at V3 growth stage was taken from both Fe zero (Fe^-^) and Fe added (Fe^+^) conditions. Green and red color represents down-regulation, and up-regulation in the color scale, respectively.

Genes involved in Fe metabolism of six chickpea genotypes grown under Fe zero (Fe^-^) and Fe added (Fe^+^) conditions at V3 growth stage are presented in **Figure 5**.

Two Fe uptake genes *FRO2* and *IRT1* expressed differently across the six chickpea genotypes (**Figure 5**). At V3 growth stage, under Fe added (Fe^+^) condition, *FRO2* was expressed more in root tissues than in leaves compared to Fe zero (Fe^-^) (**Figures 5A, E**). Among the cultivated genotypes, genotype CDC 551-1 showed the highest level of expression of *FRO2* under Fe added condition in roots, which is 14, 12, and 10-folds higher than the *FRO2* expression in wild genotypes Kalka 064, Sarik 067, and Cermi 075, respectively. Like CDC 551-1, the cultivated genotype FLIP97-677C also showed high expression of *FRO2* under added Fe condition in roots, which is 9, 8 and 6-folds higher than the *FRO2* expression in wild genotypes Kalka 064, Sarik 067, and Cermi 075, respectively (**Figure 5A**). However, in leaves, most of the genotypes showed lower expression of *FRO2* under both Fe added and Fe zero conditions (**Figure 5E**). Another Fe regulated gene in roots, *IRT1*, showed higher expression in leaves (**Figure 5F**) rather than in roots (**Figure 5B**). The highest level of *IRT1* expression was observed in Cermi 075 under Fe zero condition in leaves, whereas the lowest *IRT1* expression was shown by FLIP97-677C and Sarik 067 (**Figure 5F**). Our findings also showed that under Fe zero condition, the expression of *IRT1* was higher in most of the genotypes in both leaves (**Figure 5F**) and roots (**Figure 5B**) compared to Fe added condition.

**Figure 6:**
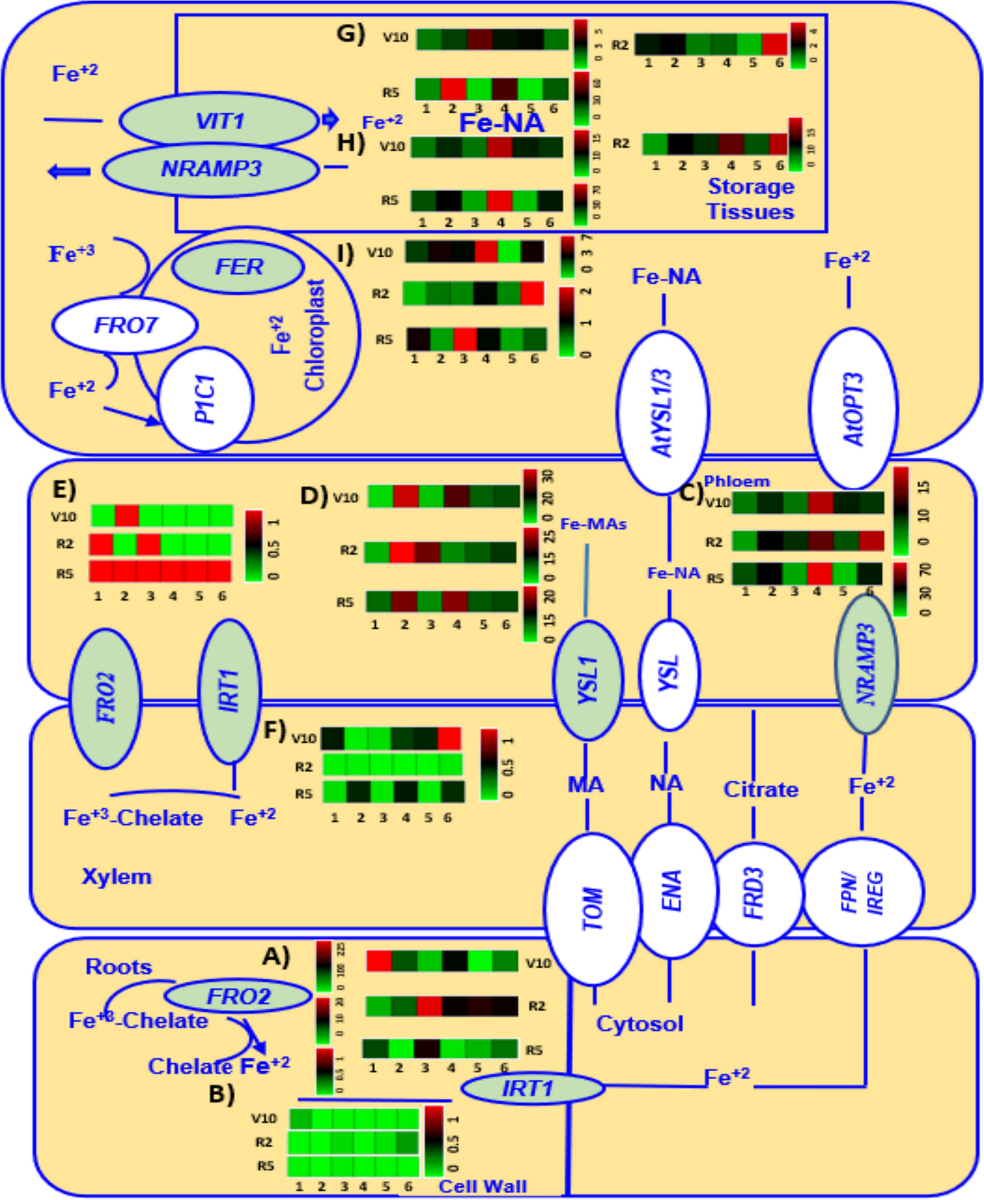
A heatmap analysis showing the gene expression patterns of Fe metabolism related genes *FRO2* **(A, E)**, *IRT1* **(B, F)**, *NRAMP3* **(C, H)**, *YSL1* **(D)**, *V1T1* **(G)**, and *FER3* **(I)** in roots and leaves of six different genotypes (1 = CDC Verano, 2 = Cermi 075, 3 = FLIP97-677C, 4 = Sarik 067, 5 = Kalka 064, and 6 = CDC 551-1). The data at V10, R2 and R5 growth stages taken only from Fe added (Fe^+^) conditions. Green and red color represents down-regulation, and up-regulation in the color scale, respectively.

Three selected Fe transporter genes: *NRAMP3* (**Figures 5C, H**)*, V1T1* (**Figure 5G**)*, and YSL1* (**Figure 5D**) were mainly expressed in leaves compared to roots (**Supplementary Figure S1**). Under Fe zero condition, an increase of *NRAMP3* expression was observed in leaves of the six genotypes compared to Fe added condition (**Figures 5C** and **6H**). In roots, the *NRAMP3* showed lower expression in all six genotypes under both conditions compared to leaves. However, the expression levels of *NRAMP3* were relatively high in most genotypes except CDC Verano and Cermi 075 under Fe zero condition compared to Fe added condition (**Supplementary Figure S1**). Gene *V1T1* expressed more in leaves under Fe added condition in all six genotypes (**Figure 5G**). Under Fe added condition the highest level of *V1T1* expression (5 folds) was obtained in the leaves of Cermi 075 compared to Fe zero (**Figure 5G**). We also found that under Fe deficient condition higher level of *YSL1* expression compared to Fe added conditions in all genotypes except CDC Verano and FLIP97-677C (**Figure 5D**). However, in roots, we found that the expression of *YSL1* was higher under Fe added than Fe zero conditions in all genotypes except FLIP97-677C and Kalka 064 (**Supplementary Figure S1**). Moreover, leaf tissues have higher expression of *FER3* under Fe zero condition in comparison to Fe added condition, except Sarik 067 and CDC-551-1 which showed relatively high expression of *FER3* under Fe added condition (**Figure 5I**).

At V3 stage, we found that the *WEE1* gene was up regulated in both tissues when the plants were subjected to Fe zero condition (**Supplementary Figure S1**). In the current study at V3 stage, both root and leaf tissues showed elevated expression of *WEE1* and *GCN2* genes when the plants were subjected to Fe zero environment (**Supplementary Figure S1**).

#### 3.5.2 V10, R2, and R5 growth stage

Expression of genes (highlighted in green) involved in Fe metabolism (*FRO2*, *IRT1*, *NRAMP3*, *V1T1*, *YSL1, FER3*, *WEE1*, and *GCN2)* of six chickpea genotypes grown under Fe added condition at V10, R2, and R5 growth stages are presented in **Figure 6**.

In the current study expression of Fe uptake genes, *FRO2* (**Figures 6A, E**) and *IRT1*(**Figures 6B, F**), was higher in roots (**Figures 6A, B**) compared to leaves (**Figures 6E, F**) across all six genotypes (**Figure 6**) at V10, R2, and R5 stages. The highest level of expression of *FRO2* was observed in V10 roots compared to R2 and R5 stages across all six genotypes (**Figure 6A**). Unlike *FRO2*, expression of *IRT1* was highest in V10 leaves among all other stages in root and leaf tissues (**Figures 6B, F**).

This study also found the highest expression of *NRAMP3*(**Figures 6C, H**), and *VIT1*(**Figure 6G**) in R5 leaves compared to V10 and R2 leaves except Kalka 064 (**Figures 6C, H, G**). Like in *NRAMP3* and *VIT1*, the highest expression of *YSL1* was also found in R5 leaves except in leaves of Kalka 064 and FLIP97-677C (**Figure 6D**). However, at V10 stage, we found that the expression of both *NRAMP3* (**Figures 6C, H**), and *YSL1*(**Figures 6B, F**) was higher in leaves (**Figure 6**) compared to roots (**Supplementary Figure S2**) except CDC Verano and CDC 551-1. However, at R2, roots showed higher expression of *NRAMP3* than leaves except Cermi -075. Similarly, at R2, the higher expression of *YSL1* was also found in roots compared to leaves except Cermi -075, FLIP97-677C, and CDC-551-1. At R5, leaves showed higher expression of *NRAMP3* and *YSL1* than roots except FLIP97-677C and Kalka 064 of *NRAMP3* and FLIP97-677C of *YSL1* (**Figure 6** and **Supplementary Figure S2**). However, the higher expression of *V1T1* was found in leaves compared to roots across all genotypes at V10 to R5 stages except Kalka 064 at R2 (**Figure 6G** and **Supplementary Figure S2**).

In this study, we also found that roots showed higher expression of *FER3* compared to leaves across all genotypes and stages except Kalka 064 at V10 leaf tissues (**Figure 6I** and **Supplementary Figure S2**). Like in *FER3*, the expression of WEE1 was higher in roots compared to leaves across all genotypes and stages except FLIP97-677C and Kalka 064 at R5 roots (**Supplementary Figure S2**). Another gene involved in Fe metabolism, *GCN2*, expressed more in roots compared to leaves across all genotypes and growth stages. In addition, at V10 and R2 stages both in root and shoot tissues compared to R5 (**Supplementary Figure S2**).

### 3.6 Correlation study of the gene expressions and Fe concentrations

The correlation between the Fe concentration and the gene expression in roots and leaves at V3 to R5 growth stages of the six chickpea genotypes grown under Fe zero and Fe added conditions are shown in **Table 4**.

**Table 4:** Correlation between Fe concentration and gene expression in roots and leaves at V3 to R5 growth stage of six genotypes grown under Fe zero and Fe added conditions.

	Roots					Leaves				
	Fe zero	Fe added				Fe zero	Fe added			
Genes	V3	V3	V10	R2	R5	V3	V3	V10	R2	R5
*FRO2*	ns	0.95**	0.87*	ns	ns	ns	ns	0.82*	ns	ns
*IRT1*	ns	0.99**	0.99**	0.87*	ns	ns	ns	ns	ns	ns
*NRAMP3*	ns	ns	0.95**	ns	ns	ns	ns	ns	ns	ns
*VIT1*	ns	ns	ns	ns	ns	ns	0.88*	ns	ns	ns
*YSL1*	ns	0.87*	ns	ns	ns	ns	ns	ns	ns	ns
*FER3*	ns	ns	0.88*	ns	ns	ns	ns	ns	ns	ns
*GCN2*	0.81*	ns	ns	ns	ns	ns	0.86*	ns	ns	ns
*WEE1*	ns	ns	ns	ns	ns	ns	ns	ns	ns	ns

ns, non-significant; * = significant at p < 0.05; and ** = significant at p < 0.01.

Among the selected growth stages, under Fe added condition in roots, the relationship between the Fe concentration and *FRO2* showed a strong positive correlation (r = 0.95; *p* < 0.01) at V3, and (r = 0.86; *p* < 0.05) at V10, respectively (**Table 4**). Similarly, under Fe added condition in leaves, *FRO2* showed a strong positive correlation (r = 0.82; *p* < 0.05) at V10 in leaves (**Table 4**). Like *FRO2*, under Fe added medium, *IRT1* also showed a strong positive correlation (r = 0.99; *p* < 0.01) at V3, (r = 0.99; *p* < 0.01) at V10, and (r = 0.87; *p* < 0.05) at R2 growth stages, respectively (**Table 4**). Likewise, under Fe added environment in roots, *NRAMP3, YSL1, FER3*, and *GCN2* showed a strong positive correlation (r = 0.95; *p* < 0.01) at V10, (r = 0.87; *p* < 0.05) at V3, (r = 0.88; *p* < 0.01) at V10, and (r = 0.81; *p* < 0.05) at V3, respectively (**Table 4**). Moreover, under Fe zero condition in leaves, *FRO2, V1T1*, and *GCN2* showed a strong positive correlation (r = 0.82; *p* < 0.05) at V10, (r = 0.88; *p* < 0.05) at V3, and (r = 0.86; *p* < 0.05) at V3, respectively (**Table 4**).

## 4 Discussions

A better understanding of the mechanism of Fe accumulation in seeds is a prerequisite for developing chickpea cultivars with high Fe amount in seeds. Although major advances have been made in generating engineered biofortified crops, the Fe loading pathway into seeds is still unclear (Grillet et al., 2014). To enhance our knowledge in this regard, a study was conducted to evaluate Fe accumulation in organs at different growth stages of chickpea. Our results showed that Fe was remobilized from roots to the sink tissues under Fe zero environment are consistent with the previous studies done in wheat, *Arabidopsis thaliana*, and chickpea (Mahmoudi et al., 2005; Peng and Li, 2005; Waters and Grusak, 2008; Waters et al., 2009; Sankaran and Grusak, 2014). Our Fe accumulation study also showed more Fe remobilization under Fe zero condition compared to Fe added condition. Also, our findings showed that Fe concentration level in seeds was higher in reproductive stages (R5 and R6) compared to full maturity stage (RH). This finding suggests that at the reproductive stage Fe remobilize more from the other tissues to the seeds than at physiological maturity stage, which is consistent with previous studies of wheat, and *Arabidopsis thaliana* (Himelblau and Amasino, 2001; Garnett and Graham, 2005).

In this study, we also measured Fe amount which is a better measurement option for selecting cultivars for Fe improvement than is Fe concentration. This is because Fe amount allow to assess total Fe accumulation as well as Fe remobilization in different organs. Our findings showed that the Fe amount in root, stem, and leaf increased gradually over the six time points suggests that little or no Fe remobilization occurred at the later growth stages, which is consistent with the results in the previous study in pea (Sankaran and Grusak, 2014). We also found that Fe amount in seeds was higher at physiological maturity stage (RH) compared to reproductive (R5 and R6) stages suggested that at reproductive stages, total incoming Fe either from continuous uptake or remobilization from other tissues might be distributed to new leaves, roots, stems, flowers, and seeds. However, at physiological maturity stage (RH), under Fe added condition, continuous uptake of Fe replaced remobilization that ultimately help to increase seed Fe accumulation. Similar results have been reported in wheat, *Arabidopsis thaliana*, and pea (Himelblau and Amasino, 2001; Garnett and Graham, 2005; Sankaran and Grusak, 2014).

This study also observed that under Fe added condition, the cultivated genotypes (such as CDC Verano, FLIP97-677C and CDC 551-1) contained higher Fe amount in roots than the wild genotypes (**Supplementary Table S4**). However, in seeds, the higher Fe amount was found in the three selected wild genotypes Sarik 067, Cermi 075, and Kalka 064 than the selected cultivated species FLIP97677C, CDC-551-1, and CDC Verano (**Figures 4A, B**). Therefore, our results indicated that Fe absorption and accumulation rate also depend on cultivars, which is consistent with previous studies in common bean and rice (Blair et al., 2010; Pereira et al., 2014).

In this study, we also measured root and shoot dry weight as well as root to shoot ratio under Fe zero and Fe added conditions at V3 and V10 growth stages (**Table 3**). Our findings showed that under Fe added conditions both root and shoot dry weight as well as root to shoot ratio was higher compared to Fe zero conditions at both V3 and V10 growth stages. This study also observed that shoot dry weight under both Fe zero and Fe added conditions was higher than root dry weight at both V3 and V10 growth stages. Our findings indicated that Fe zero condition reduced both root and shoot dry weight compared to Fe added conditions at both V3 and V10 growth stages. In addition, root dry weight was lower than shoot dry weight at both conditions and growth stages. Similar observations were reported in lentils, chickpeas, and soybean (Mahmoudi et al., 2005; Vasconcelos et al., 2006).

Another objective of this study was to determine the relative expression levels of Fe-related transporter and Fe metabolism genes in chickpea seeds. For this, a study was conducted to evaluate the expression levels of genes associated with Fe uptake, transportation, and accumulation into the seeds of chickpea. We analysed gene expression of selected genes for six chickpea genotypes grown under Fe zero and Fe added conditions at V3 (**Figure 5**), V10, R2 and R5 stages (**Figure 6**) under Fe added condition. Fe uptake gene *FRO2* generally expressed more under Fe zero environment. However, here, both at V3 and V10 to R5 growth stages, our study showed *FRO2* expressed more in roots than leaves under Fe added condition, which is consistent with the previous studies in soybean and common bean (Blair et al., 2010; Santos et al., 2013). In the present study, under Fe added condition at V3 in roots, we also observed genotype CDC 551-1 showed the highest level of expression among all selected genotypes (**Figure 5A**). Consistent with our results previous reports showed that the activity of *FRO2* depends on both species and cultivar (Blair et al., 2010; Santos et al., 2013; Pereira et al., 2014). However, under Fe zero condition at V3, another Fe regulates gene, *IRT1*, showed higher expression in leaves as well as root tissue (**Figures 5B, D**). However, under Fe added condition, we found higher expression of *IRT1* mostly in leaves of six genotypes compared to roots except R2 (**Figures 6B, F**). Although *IRT1* express more under Fe zero condition, a previous study in *Arabidopsis thaliana* showed that the regulation of *IRT1* depends on the root Fe status and shoot Fe demands (Vert et al., 2003). Moreover, as *IRT1* belongs to the ZIPs family that are not only involved in Fe uptake and transport Fe to the other plant organs, but also stores and detoxifies excess Fe (Grotz et al., 1998; Yang et al., 2009; Li et al., 2013).

In the present study, we also found three selected Fe transporter genes (*NRAMP3, V1T1, and YSL1)* were mainly expressed in leaves compared to roots (**Figures 5** and **6**). Our study showed under Fe zero condition, at V3 growth stage, an increase of *NRAMP3* expression was observed in leaves of the six genotypes compared to under Fe added condition (**Figures 5C, D**). In roots, the expression levels of *NRAMP3* were relatively low in all six genotypes under both Fe zero (Fe zero) and Fe added conditions (**Supplementary Figure S1**). Like in V3, at V10 -R5 stages, a higher expression of *NRAMP3* was found in leaves of most of the selected genotypes compared to roots under Fe added condition (**Figures 6C, H**). These results supported the fact that this gene is mainly predominant in leaves (**Figures 5C, H** and **6C, H**). The conclusion that emerged from this study is similar to that in *Arabidopsis thaliana* reported by Lanquar et al. (2010). Gene *V1T1* is responsible for Fe loading into the vacuole, which is completely the opposite role of *NRAMP3*. Thus, it is expected that this gene would be expressed more under Fe added leaves as shown in all six genotypes at V3 and V10 - R5 as well. Our results also showed both at V3 and V10 - R5 stages, gene *V1T1* expressed more under Fe added shoot as shown in all six genotypes at V3 and V10 to R5 compared to roots (**Figures 5G** and **6G**, **Supplementary Figures S1, S2**). These findings are consistent with previous studies in which the expression of *V1T1* increased in parallel with the accumulation of Fe^2+^ into the vacuoles, to lessen waste, and to cut down the oxidative damage (Bakhshi and Karimian, 2004).

Like the other two transporter genes *NRAMP3* and *V1T1*, *YSL1* also transports Fe in the form of ${\text{Fe}}_{2}^{+}$ NA complexes, which is the crucial transportable Fe form in the phloem (Kim et al., 2006). Thus, it is expected that this gene will be expressed more under Fe added condition. This is because under Fe added condition Fe transport activity is needed to fulfil plant requirement. Our findings also found that the root tissues at V3 stage under Fe-treated condition showed higher level of *YSL1* expression compared to Fe zero (Fe zero) conditions (**Supplementary Figure S1**). These findings support the previous reports that Fe was translocated to various plant organs (Kim et al., 2006). Similar observation was also found at V10 - R5 stages under Fe added condition (**Figure 6D**). However, other findings from this study showed that the *YSL1* expression in shoot tissues was higher in plants grown under Fe zero than Fe added condition at V3 stage (**Figure 5D**), which is in contrast with previous study in *Arabidopsis thaliana* (Kim et al., 2006).

Ferritin, which is the Fe storage protein, provides insurance for meeting Fe demands of cell without reacting with oxygen when Fe is in affluent (Briat et al., 2010). In this study, at V3 stage, leaf tissues under Fe zero condition showed higher level of *FER3* expression compared to leaf under Fe-available condition, except for the CDC-551-1 (**Figure 5I**). The conclusion that emerged from this study is contrast to that in (Ravet et al., 2009). However, at V10 – R5 stages, our study found at V10 stage leaves showed an increase level of *FER3* relative expression compared to leaves at flowering (R2) and physiological maturity stage (R5). This process led the movement of the cellular Fe demand from source (leaf tissues) to other parts of plants (flowers and seeds) at R2 and R5 stages (**Figure 6I**) when the plants were under Fe added condition. The conclusion that emerged from this study is similar to that in (Ravet et al., 2009)

In this study, other two genes: *WEE1* and *GCN2*, which are responsible for Fe metabolism were also studied. *WEE1* is a protein that is associated with cell cycle regulation (de Schutter et al., 2007). Thus, the absence of *WEE1* function led to stunted plant growth (Jones et al., 2013). At V3 stage, we found higher expression of *WEE1* and *GCN2* in both root and leaf tissues of plants grown under Fe zero condition than under Fe added condition (**Supplementary Figure S1**). However, under Fe added condition both at V3 and V10 to R5 stages, the expression of *WEE1* and *GCN2* were observed in roots compared to leaves (**Supplementary Figures S1 and S2**). Our findings were previously reported in *G. max* and *M. truncatula* (Mendes, 2014).

In the current study, we also observed the correlations between the Fe concentrations and the gene expressions in roots and leaves at V3 to R5 growth stages of the six chickpea genotypes grown under Fe zero and Fe added media (**Table 4**). At V3 growth stage under Fe added medium, we found genes *FRO2*, showed a highly significant positive correlation at V3 and V10 in roots, and at V10 in leaves, respectively, with Fe concentration (**Table 4**). Although *FRO2* is needed to reduce ferric to ferrous Fe mainly in the roots under Fe zero condition, current findings observed *FRO2* expressed more under Fe added condition. This finding suggests that the *FRO2* requires more Fe to activate itself during the Fe zero condition. As a result, *FRO2* expression reduced during Fe zero condition. In addition, higher expression of *FRO2* helps to increase more available ferrous Fe. Thus, the large amount of Fe is stored in roots and leaves under Fe available environment. Previous studies conducted in common bean, soybean, and *Medicago truncatula* reported that an elevated *FRO2* expression increased Fe concentration in roots and leaves (Blair et al., 2010; Mendes, 2014). Current findings with chickpea provided further confirmation of these reports. Like *FRO2*, under Fe added medium, *IRT1* also showed a strong positive correlation at V3, V10, and R2 growth stages, respectfully (**Table 4**). A study in pea reported that *FRO2* is the rate limiting enzyme for Fe acquisition, Fe transporter gene *IRT1* cannot achieve saturation level of Fe concentration without the Fe reductase activity (Grusak et al., 1990). This suggests that *FRO2* and *IRT1* are coregulated. Previous studies in soybean and medicago truncatula, also reported that the level of *IRT1* expression is equivalent with *FRO2* expression in both species and tissues (Mendes, 2014). Similar observation was also found in *Arabidopsis thaliana* (Kim and Guerinot, 2007). Our findings were consistent with these reports.

Likewise, under Fe added environment in roots, *NRAMP3*, and *YSL1* showed a strong positive correlation. Although the role of *NRAMP3* is to remobilize Fe from the vacuole, *NRAMP3* express more in roots at V10 growth stage along with Fe concentration under Fe available media. The reason behind this finding is that under Fe added medium, plants do not require more Fe to be remobilized to the leaves, which is contrast to Fe zero condition. Thus, higher level of *NRAMP3* expression was found along with Fe concentration in roots under Fe available media. This finding is in contrast with reports on *Arabidopsis thaliana*, soybean and *Medicago truncatula* where *NRAMP3* express more in leaves under Fe zero media (Lanquar et al., 2010; Mendes, 2014). Since *YSL1* transport Fe^2+^-NA complex, which is the main transportable Fe form in the phloem, it is expected to increase more transporter like *YSL1* to fulfil plant’s demand under Fe available media. This finding agrees with reports on soybean and medicago where *YSL1* expression increase more in both tissues along with Fe concentration under Fe added condition (Kim et al., 2006; Mendes, 2014). Current work also found that a highly significant positive correlation between the Fe concentration and *V1T1* expression at V3 under Fe added environment in leaf (**Table 4**). Since gene *V1T1* plays the opposite role of *NRAMP3*, which is Fe loading into the vacuole, it is expected that this gene would be expressed more under Fe added leaves. The findings from this study is similar to that in (Bakhshi and Karimian, 2004).

The result from the present work also obtained that a highly significant positive correlation was detected between the Fe concentration and *FER3* expression in roots at V10 under Fe added condition (**Table 4**). This finding matches with reports on *Arabidopsis thaliana* where *FER3* express more with Fe concentration under Fe added condition (Ravet et al., 2009). We also found a strong positive correlation between the Fe concentration and *GCN2* expression in roots and leaves at V3 under Fe zero and Fe added conditions, respectively (**Table 4**). Our findings that *GCN2* plays a significant role in Fe metabolism were previously reported in *G. max* and *M. truncatula* (Mendes, 2014).

In conclusions, results from our present study indicates that the genes responsible for Fe uptake and translocation such as *FRO2*, *IRT1*, *NRAMP3*, *VITI*, and *YSL1*, as well as Fe storage (*FER3*) and Fe metabolism genes (*GCN2* and *WEE1*), can be targeted for selection and modification to increase Fe concentration in chickpea seeds. Moreover, in terms of Fe improvement in chickpea seeds, wild species could be a better option than cultivated species.

## Data availability statement

The original contributions presented in the study are included in the article/**Supplementary Material**. Further inquiries can be directed to the corresponding author.

## Author contributions

TJ and BT designed the experiment. TJ conducted the experiments, analysed the data, and prepared the draft manuscript. SK and BT assisted with data analysis and edited the manuscript. BT conceived and directed the research. All authors reviewed all documents critically and approved the final manuscript for submission in the journal. All authors contributed to the article and approved the submitted version.
