# Supplementary material for: Iron accumulation and partitioning in hydroponically grown wild and cultivated chickpea (Cicer arietinum L)
Source: Front Plant Sci. 2023 Mar 17;14:1092493. doi: 10.3389/fpls.2023.1092493 (PMC10063876; doi:10.3389/fpls.2023.1092493)
Supplement: Supplementary file 3 [file Table_1.docx]

**Supplementary Table S1.** Mean Fe concentration (µg g^−1^, ± SE; n = 8) at R2, R5, R6, and RH stages in roots of six chickpea genotypes grown under hydroponic system.

| Genotype | Growth stage | Fe concentration (µg g^−1^,  ± SE)  in roots |
| --- | --- | --- |
| CDC-551-1 | R2 | 1222 (±10.4) |
| (*C. arietinum*) | R5 | 686 (±8.5.0) |
|  | R6 | 1232 (±17.6) |
|  | RH | 1782 (±18.0) |
| CDC Verano | R2 | 375 (±3.2) |
| *(C. arietinum)* | R5 | 403 (±9.0) |
|  | R6 | 1062 (±15.0) |
|  | RH | 2516 (±25.0) |
| FLIP97-677C | R2 | 1002(±9.0) |
| *(C. arietinum)* | R5 | 588(±6.0) |
|  | R6 | 644(±14.0) |
|  | RH | 1903(±23.4) |
| Kalka 064 | R2 | 610 (±4.0) |
| *(C. reticulatum)* | R5 | 553 (±4.0) |
|  | R6 | 1440 (±13.0) |
|  | RH | 1386 (±25.0) |
| Sarik 067 | R2 | 530 (±3.8) |
| *(C. reticulatum)* | R5 | 413 (±10.8) |
|  | R6 | 1400 (±17.9) |
|  | RH | 1316 (±29.0) |
| Cermi 075 | R2 | 701 (±8.4) |
| *(C. echinospermum)* | R5 | 500 (±8.7) |
|  | R6 | 1105 (±18.5) |
|  | RH | 1487 (±29.7) |
