# Supplementary material for: Iron accumulation and partitioning in hydroponically grown wild and cultivated chickpea (Cicer arietinum L)
Source: Front Plant Sci. 2023 Mar 17;14:1092493. doi: 10.3389/fpls.2023.1092493 (PMC10063876; doi:10.3389/fpls.2023.1092493)
Supplement: Supplementary file 4 [file Table_2.docx]

**Supplementary Table S2.** Mean Fe concentration (µg g^−1^, ± SE; n = 8) at R2, R5, R6, and RH stages in stems of six chickpea genotypes grown under hydroponic system.

| Genotype | Growth stage | Fe concentration (µg g^−1^,  ± SE) in stems |
| --- | --- | --- |
| CDC-551-1 | R2 | 25 (±2.8) |
| (*C. arietinum*) | R5 | 14 (±0.3) |
|  | R6 | 17(±0.9) |
|  | RH | 16(±2.5) |
| CDC Verano | R2 | 21 (±0.4) |
| *(C. arietinum)* | R5 | 15 (±0.9) |
|  | R6 | 21 (±1.5) |
|  | RH | 18 (±1.5) |
| FLIP97-677C | R2 | 26 (±0.53) |
| *(C. arietinum)* | R5 | 20 (±1.5) |
|  | R6 | 22 (±1.0) |
|  | RH | 24 (±1.1) |
| Kalka 064 | R2 | 18 (±2.5) |
| *(C. reticulatum)* | R5 | 16 (±1.0) |
|  | R6 | 17 (±1.5) |
|  | RH | 19 (±1.3) |
| Sarik 067 | R2 | 20 (±2.8) |
| *(C. reticulatum)* | R5 | 21 (±0.7) |
|  | R6 | 13 (±1.2) |
|  | RH | 19 (±0.5) |
| Cermi 075 | R2 | 24(±0.9) |
| *(C. echinospermum)* | R5 | 22(±2.1) |
|  | R6 | 19(±0.8) |
|  | RH | 21(±0.9) |
