# Supplementary material for: Iron accumulation and partitioning in hydroponically grown wild and cultivated chickpea (Cicer arietinum L)
Source: Front Plant Sci. 2023 Mar 17;14:1092493. doi: 10.3389/fpls.2023.1092493 (PMC10063876; doi:10.3389/fpls.2023.1092493)
Supplement: Supplementary file 5 [file Table_3.docx]

**Supplementary Table S3.** Mean Fe concentration (µg g^−1^, ± SE; n = 8) at R2, R5, R6, and RH stages in leaves of six chickpea genotypes grown under hydroponic system.

| Genotype | Growth stage | Fe concentration (µg g^−1^,  ± SE) in leaves |
| --- | --- | --- |
| CDC-551-1 | R2 | 71 (±5.9) |
| (*C. arietinum*) | R5 | 77 (±2.2) |
|  | R6 | 80 (±5.5) |
|  | RH | 50 (±3.2) |
| CDC Verano | R2 | 66 (±3.0) |
| *(C. arietinum)* | R5 | 95 (±5.4) |
|  | R6 | 83 (±3.1) |
|  | RH | 72 (±0.7) |
| FLIP97-677C | R2 | 105 (±1.2) |
| *(C. arietinum)* | R5 | 90 (±2.3) |
|  | R6 | 99 (±1.8) |
|  | RH | 82 (±1.4) |
| Kalka 064 | R2 | 85(±6.2) |
| *(C. reticulatum)* | R5 | 90(±2.1) |
|  | R6 | 83(±1.2) |
|  | RH | 75(±1.6) |
| Sarik 067 | R2 | 94 (±2.0) |
| *(C. reticulatum)* | R5 | 101 (±2.1) |
|  | R6 | 81 (±4.5) |
|  | RH | 73 (±3.2) |
| Cermi 075 | R2 | 103 (±3.1) |
| *(C. echinospermum)* | R5 | 113 (±0.5) |
|  | R6 | 84 (±1.2) |
|  | RH | 77 (±4.2) |
