# Supplementary material for: Iron accumulation and partitioning in hydroponically grown wild and cultivated chickpea (Cicer arietinum L)
Source: Front Plant Sci. 2023 Mar 17;14:1092493. doi: 10.3389/fpls.2023.1092493 (PMC10063876; doi:10.3389/fpls.2023.1092493)
Supplement: Supplementary file 6 [file Table_4.docx]

**Supplementary Table S4.** Mean Fe amount (g, ± SE; n = 8) at R2, R5, R6, and RH stages in roots of six chickpea genotypes grown under hydroponic system.

| Genotype | Growth stage | Fe amount (g,  ± SE) in roots |
| --- | --- | --- |
| CDC-551-1 | R2 | 8.34 (±0.4) |
| (*C. arietinum*) | R5 | 9.40 (±0.7) |
|  | R6 | 22.78(±1.2) |
|  | RH | 38.81(±4.2) |
| CDC Verano | R2 | 2.40(±0.1) |
| *(C. arietinum)* | R5 | 5.40(±0.4) |
|  | R6 | 17.22(±1.0) |
|  | RH | 53.72(±1.3) |
| FLIP97-677C | R2 | 6.90(±0.4) |
| *(C. arietinum)* | R5 | 7.38(±0.8) |
|  | R6 | 11.12(±0.8) |
|  | RH | 37.88(±0.5) |
| Kalka 064 | R2 | 4.55(±0.1) |
| *(C. reticulatum)* | R5 | 7.61(±0.5) |
|  | R6 | 26.98(±0.6) |
|  | RH | 31.19(±0.9) |
| Sarik 067 | R2 | 4.49(±0.1) |
| *(C. reticulatum)* | R5 | 5.34(±0.3) |
|  | R6 | 18.50(±0.9) |
|  | RH | 22.75(±0.9) |
| Cermi 075 | R2 | 5.45(±0.3) |
| *(C. echinospermum)* | R5 | 6.47(±0.8) |
|  | R6 | 19.01(±0.7) |
|  | RH | 25.69(±1.2) |
