# Supplementary material for: Iron accumulation and partitioning in hydroponically grown wild and cultivated chickpea (Cicer arietinum L)
Source: Front Plant Sci. 2023 Mar 17;14:1092493. doi: 10.3389/fpls.2023.1092493 (PMC10063876; doi:10.3389/fpls.2023.1092493)
Supplement: Supplementary file 7 [file Table_5.docx]

**Supplementary Table S5.** Mean Fe amount (g, ± SE, n = 8) at R2, R5, R6, and RH stages in stems of six chickpea genotypes grown under hydroponic system.

| Genotype | Growth stage | Fe amount (g, ± SE)  in stems |
| --- | --- | --- |
| CDC-551-1 | R2 | 0.41(±0.0) |
| (*C. arietinum*) | R5 | 0.83(±0.0) |
|  | R6 | 1.23(±0.1) |
|  | RH | 2.08(±0.1) |
| CDC Verano | R2 | 0.58(±0.0) |
| *(C. arietinum)* | R5 | 1.06(±0.0) |
|  | R6 | 2.05(±0.1) |
|  | RH | 2.57(±0.2) |
| FLIP97-677C | R2 | 1.05(±0.0) |
| *(C. arietinum)* | R5 | 1.37(±0.1) |
|  | R6 | 2.12(±0.1) |
|  | RH | 3.49(±0.2) |
| Kalka 064 | R2 | 0.47(±0.0) |
| *(C. reticulatum)* | R5 | 1.11(±0.0) |
|  | R6 | 1.52(±0.1) |
|  | RH | 3.46(±0.1) |
| Sarik 067 | R2 | 0.36(±0.0) |
| *(C. reticulatum)* | R5 | 1.27(±0.0) |
|  | R6 | 1.32(±0.1) |
|  | RH | 1.84(±0.0) |
| Cermi 075 | R2 | 0.49(±0.0) |
| *(C. echinospermum)* | R5 | 1.55(±0.0) |
|  | R6 | 1.64(±0.0) |
|  | RH | 2.57(±0.0) |
