# Supplementary material for: Iron accumulation and partitioning in hydroponically grown wild and cultivated chickpea (Cicer arietinum L)
Source: Front Plant Sci. 2023 Mar 17;14:1092493. doi: 10.3389/fpls.2023.1092493 (PMC10063876; doi:10.3389/fpls.2023.1092493)
Supplement: Supplementary file 8 [file Table_6.docx]

**Supplementary Table S6.** Mean Fe amount (g, ± SE; n = 8) at R2, R5, R6, and RH stages in leaves of six chickpea genotypes grown under hydroponic system.

| Genotype | Growth stage | Fe amount (g, ± SE)  in  leaves |
| --- | --- | --- |
| CDC-551-1 | R2 | 0.49 (±0.0) |
| (*C. arietinum*) | R5 | 1.62(±0.1) |
|  | R6 | 2.65(±0.1) |
|  | RH | 1.12(±0.1) |
| CDC Verano | R2 | 0.47(±0.0) |
| *(C. arietinum)* | R5 | 3.62(±0.1) |
|  | R6 | 4.59(±0.1) |
|  | RH | 2.95(±0.1) |
| FLIP97-677C | R2 | 0.78(±0.0) |
| *(C. arietinum)* | R5 | 5.33(±0.1) |
|  | R6 | 6.55(±0.0) |
|  | RH | 4.54(±0.2) |
| Kalka 064 | R2 | 0.71(±0.0) |
| *(C. reticulatum)* | R5 | 4.80(±0.1) |
|  | R6 | 5.22(±0.1) |
|  | RH | 4.05(±0.1) |
| Sarik 067 | R2 | 0.77(±0.0) |
| *(C. reticulatum)* | R5 | 3.86(±0.0) |
|  | R6 | 3.67(±0.1) |
|  | RH | 2.46(±0.1) |
| Cermi 075 | R2 | 0.84(±0.0) |
| *(C. echinospermum)* | R5 | 4.75(±0.1) |
|  | R6 | 4.41(±0.1) |
|  | RH | 3.35(±0.2) |
